# What makes patients aware of their artificial knee joint?

**DOI:** 10.1186/s12891-017-1923-4

**Published:** 2018-01-08

**Authors:** F. L. Loth, M. C. Liebensteiner, J. M. Giesinger, K. Giesinger, H. R. Bliem, B. Holzner

**Affiliations:** 1 Institute of Psychology, University of Innsbruck, A-6020 Innsbruck, Austria; 2 Department for Orthopaedic Surgery, Medical University Innsbruck, A-6020 Innsbruck, Austria; 3 Innsbruck Institute of Patient-centered Outcome Research (IIPCOR), A-6020 Innsbruck, Austria; 4 Department of Orthopaedics and Traumatology, Kantonspital St. Gallen, CH-9007 St. Gallen, Switzerland; 5 Department of Psychiatry, Psychotherapy and Psychosomatics, Medical University of Innsbruck, Anichstrasse 35, A-6020 Innsbruck, Austria

**Keywords:** Arthroplasty, Osteoarthritis, Joint awareness, Total Knee Replacement (TKA)

## Abstract

**Background:**

Joint awareness was recently introduced as a new concept for outcome assessment after total knee arthroplasty (TKA). Findings from qualitative and psychometric studies suggest that joint awareness is a distinct concept especially relevant to patients with good surgical outcome and patients at late follow-up time points. The aim of this study was to improve the understanding of the concept of joint awareness by identifying situations in which patients are aware of their artificial knee joint and to investigate what bodily sensations and psychological factors raise a patient’s awareness of her/his knee. In addition, we evaluated the relative importance of patient-reported outcome parameters that are commonly assessed in orthopaedics.

**Methods:**

Qualitative interviews were conducted with patients being at least 12 months after TKA. The interviews focused on when, where and for what reasons patients were aware of their artificial knee joint. To evaluate the relative importance of ‘joint awareness’ after TKA among nine commonly assessed outcome parameters (e.g. pain or stiffness), we collected importance ratings (‘0’ indicating no importance at all and ‘10’ indicating high importance).

**Results:**

We conducted interviews with 40 TKA patients (mean age 69.0 years; 65.0% female). Joint awareness was found to be frequently triggered by kneeling on the floor (30%), climbing stairs (25%), and starting up after resting (25%). Patients reported joint awareness to be related to activities of daily living (68%), specific movements (60%), or meteoropathy (18%). Sensations causing joint awareness included pain (45%) or stiffness (15%). Psychological factors raising a patient’s awareness of his/her knee comprised for example feelings of insecurity (15%), and fears related to revision surgeries, inflammations or recurring pain (8%). Patients’ importance ratings of outcome parameters were generally high and did not allow differentiating clearly among them.

**Conclusions:**

We have identified a wide range of situations, activities, movements and psychological factors contributing to patients’ awareness of their artificial knee joints. This improves the understanding of the concept of joint awareness and of a patient’s perception of his/her artificial knee joint. The diversity of sensations and factors raising patient’s awareness of their joint encourages taking a broader perspective on outcome after TKA.

## Background

Joint arthroplasty surgery has proven to be successful in relieving pain and improving function in patients with osteoarthritis [1–3]. Traditional rating systems assessing the outcome after joint arthroplasty frequently focus on objective parameters such as range of motion and strength or on clinician ratings on function and pain. However, patients’ concerns after arthroplasty may differ significantly from those of their clinicians and they often underappreciate patients’ needs and views [4, 5]. Consequently, there has been a growing recognition that the evaluation of surgical procedures should include patient-reported outcome (PRO) instruments. These provide a more patient-centred view on treatment outcome and are becoming today’s standard [6]. This is also reflected by the fact that various countries (e.g. UK in 2009 [7], Sweden in 2002 [8], Tyrol (Austria) in 2004 [9] and Switzerland in 2012 [10]) have added mandatory PROs to their national joint replacement registries and that an increasing number of studies employ PROs as primary endpoint. However, there is still an ongoing debate with regard to the content and the assessed outcome domains depending on the targeted patient groups [6]. As joint arthroplasty has evolved and outcome has improved considerably in the last decades, commonly applied PRO tools show now relevant ceiling effects at follow-up [11–16]. This means that these tools (e.g. Western Ontario and McMaster Universities (WOMAC) Osteoarthritis Index [17], Oxford Hip Score [18, 19]) have difficulties in discriminating between patients with good outcome and patients with very good and excellent outcome.

To overcome this problem joint awareness was established as a new, more discerning construct for outcome assessment in orthopaedics. This novel construct aims to quantify the patient’s ability to completely forget about a joint in everyday life [20]. Conceptually joint awareness can be related to the theory of embodiment describing the unity of the body and the self. Awareness of a joint can be considered to result from a disunity between the affected joint and the patient’s self, whereas the forgotten joint, i.e. the patients’ ability to forget about a joint, reflects the harmony between the joint and the self, i.e. ‘cultivated immediacy’ [21]. Hudak et al. showed that in patients undergoing hand surgery the unity of the hand and the patient’s self is strongly related to patient satisfaction and the authors concluded ‘satisfaction was having a hand that could be lived with unself-consciously’ ([21] p718).

In total knee arthroplasty (TKA) the concept of joint awareness has been included in several PRO questionnaires for the assessment of surgical outcome, such as the Knee injury and Osteoarthritis Outcome Score (KOOS), the Forgotten Joint Score - 12 (FJS-12) and the Patient’s Knee Implant Performance (PKIP) questionnaire [22–24]. While these measures allow assessing the degree or frequency of awareness they do not provide a qualitative description of the patients’ experience. In addition, the relative importance of joint awareness from a patient’s perspective in comparison to other PRO parameters is unclear. Therefore, we conducted semi-structured interviews to address the following aims:

**Aim 1:** Investigation of the relative importance of commonly assessed outcome parameters

**Aim 2:** Identification of situations, activities, sensations and psychological factors that make TKA patients aware of their artificial joint

## Methods

### Sample and procedure

Patients were consecutively approached for the study if they had had a TKA at the Medical University of Innsbruck and were at least 12 months after surgery. Patients were either recruited at a follow-up visit at the department of orthopaedics or invited via mail. Inclusion criteria were:

- Any type of TKA (primary, revision, bi- and unilateral)
- Age 18–90
- No overt cognitive impairments
- Sufficient command of German
- Written informed consent

Patients recruited at the hospital first completed the FJS-12, the WOMAC and the importance ratings on a selection of outcome parameters measured by orthopaedic PRO instruments. Afterwards patients were asked to describe what makes them aware of their artificial joint within a semi-structured interview.

Patients invited via mail received a letter providing study information and an informed consent form. In addition, they received the –FJS-12, the WOMAC and the questionnaire on the importance ratings. Patients were asked to send back the questionnaires, written informed consent and their phone number and times, when they were available for a phone interview. Patients returning the questionnaires and signed informed consent were then contacted and interviewed on the phone.

For sample size determination we relied on recommendations by Morse [25] suggesting – at least 30 interviews for grounded theory approaches and on the concept of issue saturation from Glaser and Strauss [26], i.e. continuation of patient inclusion until no new issues are reported by five subsequent patients. Therefore, we planned to conduct at least 30 interviews and then continue until issue saturation was reached.

The project was approved by the local ethics committee (AN2015–0086).

### Semi-structured interview and importance ratings’

All interviews were conducted by a psychologist not involved in the treatment of the patient and who is experienced with interviewing patients for patient-reported outcome studies. The interviews were either conducted face-to-face or by phone following a standardised interview guideline.

To familiarise patients with orthopaedic patient-reported outcome measures we asked them to complete two questionnaires prior to providing the importance ratings. These two questionnaires were the Western Ontario and McMaster Universities (WOMAC) Osteoarthritis Index [17], a 24-item questionnaire assessing pain, stiffness and function, and the Forgotten Joint Score – 12 (FJS-12; [20]), a 12-item questionnaire measuring joint awareness. For the WOMAC (score range 0–100) high scores indicate poor outcome, whereas for the FJS-12 (score range 0–100) high scores indicate good outcome. For aim 1 we asked the patients to rate the importance of frequently measured outcome parameters. Based on commonly used patient-reported outcome measures (e.g. Oxford Knee and Hip Score, WOMAC, FJS-12) an interdisciplinary group of orthopaedic surgeons and psychologists selected a list of 9 parameters to be rated. These parameters were:

- Pain
- Restrictions in everyday life
- Restrictions while keeping the household
- Restrictions while doing sports
- Abnormal warm or cold feelings
- Foreign body feeling
- Stiffness
- Forget the joint in everyday life
- Trust in the joint

Importance ratings for the various outcome parameters were obtained using a rating scale with ‘0’ indicating no importance at all and ‘10’ indicating high importance.

For aim 2 patients were asked to think of situations in everyday life, when they had been aware of their artificial knee (“Please explain in your own words in which occasions or situations you get aware of your artificial joint”). Then they were asked in what way they were made aware of their knee (“Why do you get aware of your artificial knee joint?”). To ensure detailed information on occasions, situations and reasons of joint awareness, the interviewer (FLL) used clarifying questions, encouraging the patients to explain or expand their statements, or give some examples of e.g. performed activities. The interviewer took detailed notes from all patients for later analysis.

### Data analysis

Sample characteristics for sociodemographic and clinical characteristics are given as means, standard deviations and absolute and relative frequencies. For sample description we also provide means and standard deviations for the WOMAC and FJS-12 scales. For patients’ ratings for the importance of commonly assessed outcome parameters we calculated medians, means and standard deviations.

The qualitative interview data were independently reviewed by two authors (FLL and JMG). The analysis was based on the Grounded Theory approach described by Glaser [27], performing inductive coding with the help of the software NVivo 11 Pro, a software for organizing, analysing and structuring qualitative data. We first defined occasions, situations and reasons of joint awareness as theme for the analysis. Following this criterion we worked line by line though the material inductively constructing categories for each aspect fitting to the pre-defined theme. Categories could be terms (e.g. meteoropathy) or short sentences (e.g. foreign body feeling because of the implant) which characterized the material as nearly as possible. If passages fitted to the already constructed categories they were subsumed, otherwise new categories were formulated. After a first round, the category system was revised regarding the meaningfulness of the categories. In addition, we assessed if the level of abstraction was adequate to identify important occasions, situations and reasons of joint awareness. After a final work through the interviews, categories were grouped to obtain main categories (e.g. daily tasks and activities). Afterwards, categories and allocated text passages were compared and harmonized within several feedback loops between the two raters. The final category system was again applied to all interview data and codings were harmonised between the two raters. Besides examples for each category, the number of patients and the number of comments are reported as frequencies.

## Results

### Patient characteristics

We approached 44 eligible patients for study participation, 12 at a hospital visit and 32 via mail. All patients had a total knee replacement between January 2001 and June 2015. Of the 44 eligible patients four did not participate in the interview. The remaining 40 patients had an average age of 69.1 (8.8) years and 65.0% were female. The mean FJS-12 score was 46.7 (SD 28.9) and the mean WOMAC total score was 40.4 (SD 21.5). Assessment duration ranged from 20 to 45 min. Further details are given in Table 1.

**Table 1:** Patient characteristics (*n* = 40)

Age	Mean (SD)	69.1 (8.8)
Sex		N (%)
	Female	26 (65.0)
	Male	14 (35.0)
Side		
	Left	21 (52.5)
	Right	14 (35.0)
	Bilateral	5 (12.5)
Previous knee arthroplasty		
	Primary	30 (75.0)
	Revision	3 (7.5)
	Partial	2 (5.0)
	Contralateral	5 (12.5)
FJS-12	Mean (SD)	46.7 (28.9)
WOMAC	Mean (SD)	40.4 (21.5)

### Importance ratings for outcome parameters (aim 1)

All outcome parameters received a mean importance rating of at least 7.4 on the 0–10 scale (Table 2). Lowest importance was given to having no abnormal feelings (7.4 points), and having no foreign body feeling (7.9 points). Highest ratings were found for being able to trust in the joint (8.7 points), not having pain and not having restrictions in doing the household (both 8.3 points).

**Table 2:** Importance ratings of commonly assessed outcome parameters

	Median	Mean (SD)
Being able to forget the joint	10	8.1 (2.9)
No pain	10	8.3 (2.8)
No stiffness	9	8.1 (2.6)
No restrictions in everyday life	9	8.2 (2.8)
Doing sports	9	7.9 (2.4)
No restrictions doing the household	10	8.3 (2.6)
No foreign body feeling	10	7.9 (3.0)
No abnormal feelings	8	7.4 (3.0)
Trust in the joint	10	8.7 (2.5)

### Qualitative interview results for aspects of joint awareness (aim 2)

The analysis of interview data from 40 patients resulted in six main categories: activities, specific movements, pain, bodily sensations, psychological factors, and meteoropathy. Details are given in Tables 3, 4 and 5.

**Table 3:** Situations in which joint awareness occurs

Category	No. patients	%patients	No. comments
Daily tasks and activities (total)	27	67.5	95
Climbing stairs	10	25.0	23
Descending stairs	8	20.0	16
Ascending stairs	1	2.5	2
Going up or downhill	9	22.5	21
Going downhill	8	20.0	14
Going uphill	3	7.5	4
Walking	5	12.5	8
Any activity	5	12.5	6
Cleaning	2	5.0	4
Driving a car	2	5.0	4
Sleeping	2	5.0	3
House work	2	5.0	4
Gardening	1	2.5	2
Standing on a ladder	1	2.5	1
Lying	1	2.5	1
After getting up in the morning	1	2.5	1
Exercise or sports	7	17.5	16
Cycling or using an ergometer	3	7.5	6
Skiing	1	2.5	1
Running or Jogging	1	2.5	1
Squats	1	2.5	2
Specific movements (total)	24	60.0	56
Kneeling	12	30.0	20
Starting up	10	25.0	12
Extension or Flexion of the Knee	8	20.0	13
Bending over	3	7.5	4
Sitting	2	5.0	3
Standing up	1	2.5	1
Lifting the leg	1	2.5	1
Climbing on something	1	2.5	1
Meteoropathy	7	17.5	14

**Table 4:** Sensations causing joint awareness

Category	No. patients	% patients	No. comments
Bodily sensations (total)	21	52.5	62
Stiffness	6	15.0	10
No feeling or numbness	4	10.0	7
Limited flexibility	4	10.0	6
Tightness / Pulling	4	10.0	7
Swelling	4	10.0	8
Strange or unpleasant feeling	3	7.5	4
Cracking	2	5.0	4
Heat and cold sensation	2	5.0	4
Foreign Body Feeling	3	7.5	4
No strength	2	5.0	3
Rubbing	1	2.5	2
Stinging	1	2.5	2
Touch-sensitivity	1	2.5	1
Pain	18	45.0	34

**Table 5:** Psychological factors causing joint awareness

Category	No. patients	% patients	No. comments
Psychological impact (total)	9	22.5	23
Being insecure	6	15.0	10
Knowing to have artificial joint	2	5.0	2
Different from the past	1	2.5	2
Lack of pain	1	2.5	1
Fear	3	7.5	9
Fear of revision	2	5.0	3
Fear of infection or inflammation	2	5.0	4
Fear of pain	1	2.5	2

### Situations of joint awareness

#### Daily tasks and activities (95 comments from 27 patients)

Focusing on activities, patients were aware of their joints either while climbing stairs (*N* = 10; descending stairs *N* = 8), going up- or downhill (*N* = 9; going downhill *N* = 8), exercising or doing sports (*N* = 7), doing any activity (*N* = 5) and walking (*N* = 5). Sports or exercises comprised cycling, skiing, running and squats.

#### Specific movements (56 comments from 24 patients)

Regarding specific movements patients mainly were aware of their artificial joint while kneeling (*N* = 12; e.g. female, 63 years: ‘The kneecap hurts when kneeling on the floor’), starting up from resting (*N* = 10; e.g. male, 76 years: ‘after sitting for a longer time and then standing up’) and while extending and flexing of the knee (*N* = 8). Further comments included bending over (*N* = 3), sitting (*N* = 2), standing up (*N* = 1), lifting the leg (*N* = 1), and climbing on an object (*N* = 1).

#### Meteoropathy (14 comments from 7 patients)

Seven patients reported to be sensitive to changes in weather conditions or the season (e.g. female, 80 years: ‘Sometimes I got pain depending on the weather conditions. During the winter my knee is getting very cold’).

### Sensations causing joint awareness

#### Bodily sensations (62 comments from 21 patients)

Describing bodily sensations TKA patients mainly provided comments on stiffness of the knee (*N* = 6; e.g. female, 63 years: ‘In the morning I have to walk around the room so that the knee is not stiff anymore’), feelings of numbness (*N* = 4; e.g. female, 71 years: ‘lack of sensation, ‘no more strength’), limited flexibility (*N* = 4), tightness or pulling (*N* = 4), and swellings (*N* = 4; e.g. female, 60 years: ‘My knee gets swollen and hurts while doing any kind of strenuous activities’). Patients further described bodily sensations of their affected knee with a strange or unpleasant feeling (*N* = 3; e.g. male, 62 years: ‘It is a really strange feeling’), crackling (*N* = 2; e.g. male, 60 years: ‘When I move it is crackling from the kneecap up to the head’), sensation of heat or cold (*N* = 2) or having a foreign body feeling (*N* = 2; e.g. female, 47 years: ‘While walking I feel the prosthesis’).

#### Pain (34 comments from 18 patients)

Almost half of the patients (*N* = 18) provided comments related to pain. While some claimed to be in pain while kneeling (e.g. female, 81 years: ‘While kneeling - not specifically the prosthesis, but the whole knee…’ ‘If the floor is very hard…’), pain could also be triggered by meteoropathy or after resting. Some patients also reported the knee pain to have other causes than the joint arthroplasty (e.g. male, 79 years: ‘The pain might come from the hip’, ‘The pain might also emerge from the scars resulting from an accident ten years ago’).

### Psychological factors causing joint awareness (23 comments from 9 patients)

Psychological factors of joint awareness mainly comprised insecurity about the stability of the joint (*N* = 6; e.g. male, 76 years: ‘You live differently, you are more cautious, you don’t jump up - you have the impression that you have to take care of the new knee’). Patients were also afraid of having revision surgery, an infection or inflammation of the knee and of being in pain again (*N* = 3; e.g. male, 77 years: ‘I am afraid that something will happen again, that something has to be fixed again’). Two patients reported that they are aware of their artificial joint as they simply know that they do have one (*N* = 2; e.g. female, 61 years: ‘I necessarily know that I have an artificial knee’) or because it is different from the past (*N* = 1).

## Discussion

As joint awareness has gained importance as an outcome parameter in knee surgery, our study focused on providing qualitative information on the various aspects of joint awareness. In our study we identified a number of sensations, activities, situations and psychological factors that make TKA patients aware of their artificial knee joint. The most frequently found sensation causing joint awareness in our sample was pain, followed by stiffness, numbness, tightness and swelling. Among the psychological factors feelings of insecurity (e.g. related to stability) or knee-related fears were most commonly triggering awareness. The most important activities that patients reported to raise their awareness of the knee comprised descending stairs, going downhill and walking. Specific movements that were reported by patients to increase awareness were in particular kneeling, starting up, extension and flexion of the knee. In addition, meteoropathy was named as a frequent factor contributing to joint awareness.

### Limitations

Our design for evaluating the relative importance of the various outcome parameters may not have been optimal as patients had difficulties discriminating levels of importance. This resulted in rather equal importance levels across the parameters. While this may be a true finding it may also indicate that our method of assessing importance with ratings scales did not sufficiently emphasise the aspect of prioritisation. More conclusive results may have been reached by asking patients to rank the various parameters e.g. in a card sort exercise. Another limitation of our study is the sample size of 40 patients that was rather small for the quantitative analysis on importance ratings of outcome parameters. However, in the qualitative analysis we could reach issue saturation with our sample. In addition, our heterogeneous sample allowed the comprehensive identification of factors contributing to joint awareness, but the prevalence of the individual factors may not be generalizable to all TKA patients as our sample is not representative in terms of e.g. revision rates or frequency of bilateral surgery. We would like to note the high response rate in our study that was obtained through reminder calls via phone.

In a recent study [24] on the development of a PRO instrument to assess performance after TKA, patient focus groups were set up to qualitatively investigate different aspects of TKA outcome. In these focus groups joint awareness was identified as an important domain when measuring outcome after TKA. Similar to our findings the authors could demonstrate that beside the positive effects of TKA, patients noted negative changes including stiffness, numbness or tingling below the knee, meteoropathy issues, inability to kneel on the affected knee, problems going downhill or descend stairs. Furthermore the patients felt that more confidence (both physically and mentally) was needed in the knee or leg. These findings are well in line with the results from our interviews. The same study also found that patients also have distinct concepts of confidence, stability, and satisfaction when performing specific activities. This indicates that further concepts in addition to functioning or pain may have to be assessed after TKA. Joint awareness may work as an overarching parameter including several of these distinct concepts and serve as a measure of the above mentioned unselfconsciousness. The ability to forget about a joint in everyday life reflects a natural joint experience and indicates the unity of the self and the (artificial) joint. As a matter of fact this unity is not present while a patient is suffering from acute symptoms, making joint awareness a construct that gains importance at a later stage of joint recovery, i.e. after the initial rehabilitation phase and in mid- and long-term follow-up when the patient might be able to be distracted from his joint as a result of low or no symptom burden. Such a possible longitudinal shift in the relative importance of specific outcome parameters across the recovery trajectory could not be investigated within this study but, may be a worthwhile aim of future research.

## Conclusions

In conclusion, our study identified a comprehensive set of factors that make patients aware of their artificial knee. These findings help to better understand the concept of joint awareness and provide valuable information on patient’s perception of having an artificial knee joint. The heterogeneity of sensations and factors raising patient’s awareness of their joint encourages taking a broader perspective on outcome after TKA. Currently, two standardised measures [23, 24] are available that provide a score for joint awareness in patients with knee pathologies. Assessing joint awareness as a measure of the unity of the knee and the patient’s self may help to better understand patient satisfaction and distinguish surgical procedures and implants that can perform comparably when focusing only on e.g. pain, stiffness and function.

## Abbreviations

FKS-12: Forgotten Joint Score-12
KOOS: Knee injury and Osteoarthritis Outcome Score
PKIP: Patient’s Knee Implant Performance
PRO: patient-reported outcome
TKA: total knee arthroplasty
WOMAC: Western Ontario and McMaster Universities Osteoarthritis Index

## References

[1] Baker PN, van der Meulen JH, Lewsey J, Gregg PJ (2007). The role of pain and function in determining patient satisfaction after total knee replacement. Data from the National Joint Registry for England and Wales. J Bone Joint Surg Br.

[2] Bullens PH, van Loon CJ, de Waal Malefijt MC, Laan RF, Veth RP (2001). Patient satisfaction after total knee arthroplasty: a comparison between subjective and objective outcome assessments. J Arthroplast.

[3] Janse AJ, Gemke RJ, Uiterwaal CS, van der Tweel I, Kimpen JL, Sinnema G (2004). Quality of life: patients and doctors don't always agree: a meta-analysis. J Clin Epidemiol.

[4] Crane HM, Lober W, Webster E, Harrington RD, Crane PK, Davis TE, Kitahata MM (2007). Routine collection of patient-reported outcomes in an HIV clinic setting: the first 100 patients. Curr HIV Res.

[5] Kinnaman JE, Farrell AD, Bisconer SW (2006). Evaluation of the computerized assessment system for psychotherapy evaluation and research (CASPER) as a measure of treatment effectiveness with psychiatric inpatients. Assessment.

[6] Rolfson O, Eresian Chenok K, Bohm E, Lubbeke A, Denissen G, Dunn J, Lyman S, Franklin P, Dunbar M, Overgaard S (2016). Patient-reported outcome measures in arthroplasty registries. Acta Orthop.

[7] National Joint Registry for England, Wales and Northern Ireland. 12th annual report**.** [http://www.njrcentre.org.uk/njrcentre/Portals/0/Documents/England/Reports/12th%20annual%20report/NJR%20Online%20Annual%20Report%202015.pdf]. Accessed 5 Jan 2018.

[8] Garellick GKJ, Lindahl H, Malchau H, Rogmark C, Rolfson O (2014). Swedish hip Arthroplasty register, annual report 2014.

[9] Prothesenregister Tirol [https://www.iet.at/page.cfm?vpath=register/prothesenregister]. Accessed 5 Jan 2018.

[10] Swiss Arthroplasty register [http://www.siris-implant.ch/de/?id=51&L=1]. Accessed 5 Jan 2018.

[11] Wamper KE, Sierevelt IN, Poolman RW, Bhandari M, Haverkamp D (2010). The Harris hip score: do ceilingreg effects limit its usefulness in orthopedics?. Acta Orthop.

[12] Bekkers JE, de Windt TS, Raijmakers NJ, Dhert WJ, Saris DB (2009). Validation of the knee injury and osteoarthritis outcome score (KOOS) for the treatment of focal cartilage lesions. Osteoarthritis Cartilage.

[13] Thomsen MG, Husted H, Otte KS, Holm G, Troelsen A (2013). Do patients care about higher flexion in total knee arthroplasty? A randomized, controlled, double-blinded trial. BMC Musculoskelet Disord.

[14] Nilsdotter AK, Lohmander LS, Klassbo M, Roos EM (2003). Hip disability and osteoarthritis outcome score (HOOS)--validity and responsiveness in total hip replacement. BMC Musculoskelet Disord.

[15] Giesinger K, Hamilton DF, Jost B, Holzner B, Giesinger JM (2014). Comparative responsiveness of outcome measures for total knee arthroplasty. Osteoarthritis Cartilage.

[16] Hamilton DF, Giesinger JM, MacDonald DJ, Simpson AH, Howie CR, Giesinger K (2016). Responsiveness and ceiling effects of the forgotten joint Score-12 following total hip arthroplasty. Bone Joint Res.

[17] Bellamy N, Buchanan WW, Goldsmith CH, Campbell J, Stitt LW (1988). Validation study of WOMAC: a health status instrument for measuring clinically important patient relevant outcomes to antirheumatic drug therapy in patients with osteoarthritis of the hip or knee. J Rheumatol.

[18] Dawson J, Fitzpatrick R, Carr A, Murray D (1996). Questionnaire on the perceptions of patients about total hip replacement. J Bone Joint Surg Br.

[19] Dawson J, Fitzpatrick R, Murray D, Carr A (1998). Questionnaire on the perceptions of patients about total knee replacement. J Bone Joint Surg Br.

[20] Behrend H, Giesinger K, Giesinger JM, Kuster MS (2012). The “forgotten joint” as the ultimate goal in joint Arthroplasty validation of a new patient-reported outcome measure. J Arthroplast.

[21] Hudak PL, McKeever PD, Wright JG (2004). Understanding the meaning of satisfaction with treatment outcome. Med Care.

[22] Roos EM, Roos HP, Lohmander LS, Ekdahl C, Beynnon BD (1998). Knee injury and osteoarthritis outcome score (KOOS)--development of a self-administered outcome measure. J Orthop Sports Phys Ther.

[23] Behrend H, Giesinger K, Giesinger JM, Kuster MS (2012). The “forgotten joint” as the ultimate goal in joint arthroplasty: validation of a new patient-reported outcome measure. J Arthroplast.

[24] Lewis S, Price M, Dwyer KA, O'Brien S, Heekin RD, Yates PJ, Beverland D, Mordin M (2014). Development of a scale to assess performance following primary total knee arthroplasty. Value Health.

[25] Morse JM, Denizin NK, Lincoln YS (1994). Designing funded qualitative research. Handbook of qualitative research.

[26] Glaser BG, Strauss AL (1967). The discovery of grounded theory: strategies for qualitative research.

[27] Glaser B (1992). Basics of grounded theory analysis.

